# Highlights of the 1st Student Symposium of the ISCB RSG UK

**DOI:** 10.12688/f1000research.6616.1

**Published:** 2015-06-16

**Authors:** Farzana Rahman, Rohit Farmer, Sayoni Das, Fatima Vayani, Mehedi Hassan

**Affiliations:** 1 School of Computing and Mathematics, University of South Wales, Cardiff, CF37 1DL, UK; 2 School of Biosciences, University of Birmingham, UK, Birmingham, B15 2TT, UK; 3 Research Department of Structural and Molecular Biology, University College London, London, WC1E 6BT, UK; 4 Algorithms and Bioinformatics Group, King's College London, London, WC2R 2LS, UK

**Keywords:** ISCB, Regional Student groups, Bioinformatics, Life sciences

## Abstract

This short report summarises the scientific content and activities of a student-led event, the 1st student symposium by the UK Regional Student Group of the International Society for Computational Biology. The event took place on the 8th of October 2014.

## Background

The United Kingdom Regional Student Group (RSG UK) of the International Society for Computational Biology (ISCB) is a part of a global network of students and early stage post-doctoral researchers. The group was formed in December 2013, with a vision to build, strengthen and enhance a network among young computational biologists working and studying in the United Kingdom. The founders took inspirations from similar initiatives and examples set by ISCB’s student council and its regional groups ^1– 3^. The group hopes to promote the next generation of computational biologists in the UK.

Our current leadership and members include research students and postdoctoral researchers from computational biology and life sciences areas. The community offers networking, soft skill enhancement opportunities for both undergraduate and post-graduate students, and the opportunity to develop the relationship between young computational biologists and academic/industry partners within the UK.

## 1st Student Symposium on Computational Biology and Life Sciences

The inaugural event of scientific networking activity for the group was the 1st Student Symposium on Computational Biology and Life Sciences which took place on 8th October 2014. The event was held at the University of South Wales, UK. The symposium took inspirations from the workshops and conferences arranged by the ISCB Student Council and its regional student groups across the globe ^3– 5^. Fujitsu Ltd, a global provider of information technology services and HPC Wales, a pan-wales supercomputing infrastructure, supported the event.

### Scope and format of the meeting

The main scope of this meeting was to create opportunities for students and young researchers to interact with senior academics and investigators from all over the UK where they can discuss ideas, exchange knowledge and build networks. In addition, four distinguished scientists were invited to deliver keynote speeches.

The traditional scientific component of the meeting consisted of three sessions of oral presentations by students and postdoctoral researchers. The sessions also included four keynote presentations from senior scientists and one inaugural presentation on RSGs and their roles.

Dr. Rhobert Lewis from the University of South Wales gave a welcome note. Dr. Manuel Corpas, the founder of the ISCB Student Council, introduced the RSG and provided an overview of developing the next generation of computational biologists through RSGs across the globe.

Dedicated poster sessions and industrial partner talks took place during coffee breaks between oral sessions. As part of the industrial partner talk, Dr. Jonathan Mullins, from Moleculomics Ltd., gave a short presentation on generating high-value genome-scale molecular information to increase and improve knowledge of interactions between organisms and chemical compounds using the tools and services developed by Moleculomics Ltd.

The RSG UK team received more than 20 abstracts from students who wished to present their work at the symposium. The submitted abstracts were peer-reviewed by ten independent reviewers of which five reviewers were senior academic and principal investigators and other five reviewers were mid-career researchers. The reviewers selected a total of six abstracts for oral presentations, and twelve abstracts for poster presentations. Overall, 65 delegates from 24 UK institutions and industries attended the symposium and the programme.

All abstracts accepted for oral and poster presentations are available online from http://rsg-uk.iscbsc.org/sym2014/. We also hope to organise a *F1000Research* channel targeted at young researchers where these abstracts will be made available. Our delegates were encouraged to submit their presentations to F1000Research.

### List of keynote speakers

The following scientists generously agreed and delivered keynotes speeches on the day.

- Dr. Alex Bateman (EMBL-EBI, Cambridge)
- Dr. Natasha De Vere (National Botanic Garden of Wales)
- Dr. Chris Creevey (IEBRS, Aberystwyth University),
- Dr. Tatiana Tatarinova (University of Southern California)

## Highlights

### Keynotes summary

Dr. Natasha de Vere’s keynote speech gave an overview of her work on DNA Barcoding of Welsh and UK flora to create an open access resource for people, wildlife and the environment ^6^. In 2012, Dr. de Vere led a team to complete the barcoding of all of Welsh flora and is now working on barcoding the British flora.

The second keynote speech by Dr. Alex Bateman underlined the impacts of biological databases in modern-day computational biology research and highlighted the importance of the role of bio-curators in biological studies. Dr. Bateman emphasised the role of biocurators by addressing them as the “unsung heroes of biology”, who order and make sense of the immense primary literature for us. He encouraged the audience to participate in contributing to the public encyclopedia, ‘Wikipedia’, and shared his recent experiences with some initiatives to inspire the scientific community to enrich Wikipedia ^7– 9^. In the second part of his talk, he gave our delegates an overview of his career path, which included some invaluable advice for young researchers hoping to make their way into a successful research career.

The third keynote speech by Dr. Christopher Creevey highlighted the development of a system-level approaches in understanding microbial community interactions by identifying genomic factors influencing phenotypic changes in organisms from Bacteria to Eukaryotes. Dr. Creevey’s talk covered his group’s work on rumen bacterial diversity ^10^. The project surveyed the rumen bacterial diversity to examine the culturable fraction of the rumen bacterial microbiome and reported that there are few novel but many uncultured taxa within the rumen bacterial microbiome. In addition, they have identified taxa where further cultivation efforts are clearly required.

Finally, Dr. Tatiana Tatarinova’s presented two novel algorithms, GPS ^11^ and reAdmix ^12^, that were developed to determine bio-geographic origins of an individual. Both methods are species-independent and were successfully applied to the analysis of human ancestry. Dr. Tatarinova also discussed exciting aspects of a bioinformatician’s life, such as field trips to remote parts of the Earth to find nearly extinct species, studies of ancient DNA and efforts to analyze and preserve DNA of disappearing Native Siberians.

### Student and early career researchers’ presentations

There were two presentations from postdoctoral research fellows and four presentations from current PhD students. In the first postdoctoral talk, Dr. Romain Studer from EMBL-EBI, Cambridge, UK discussed his current research on finding an evolutionary pattern of the phosphoproteome in 18 yeast species. Dr. Studer highlighted that to date, the investigation of the phosphoproteome has mostly covered model species (e.g. human, baker’s yeast), and that there is a lack of comprehensive phylogenetic analysis of phosphorylation. He and his colleagues are currently working on estimating the evolutionary analysis of phosphosites in yeast species.

Dr. Ranajit Das from Sheffield University gave the second postdoctoral talk, where he discussed the evolution of miRNAs and their targets among hominoid primates. Dr. Das is working on identifying and understanding the uniquely gained and lost miRNAs within hominoids and their potential implications.

In the student presentation section, Sam Nicholls from Aberystwyth University presented Goldilocks, a Python package that provides users with functionality for locating suitable regions within a genome for analysis. The Goldilocks package is available at https://github.com/SamStudio8/goldilocks.

Francesco Rubino from Aberystwyth University gave an insight into metagenomics analysis of the rumen microbiome that reveals functional isoforms driven from niche differentiation for nutrient acquisition.

Silvia Bartolucci from King’s College London, presented a statistical approach to investigating basic, systematic features exhibited by adaptive immune systems. Silvia’s work focuses on dynamical analysis of diluted associative networks: a minimal model for the adaptive immune system.

Russel Sutherland from King’s College London presented his work on tumor grade prediction across multiple adenocarcinomas using exome sequencing data.

## Award winners

HPC Wales and Fujitsu Services UK limited sponsored two travel fellowships that were awarded to Silvia Bartolucci and Vasileios Panagiotis Lenis in order to attend and present at the Symposium.

A review panel of senior researchers present at the event evaluated all presentations and selected three speakers for awards; one for the oral presentation and two for poster presentations. Sam Nicholls from the Aberystwyth University received the best oral presentation award for his work titled “Goldilocks: Locating genomic regions that are ‘just right’”.

Daniele Avancini received best poster award for his poster titled “A Molecular Dynamic Web Server for Analysis of DNA Structure” and the runner up poster award went to Stefani Dritsa for his poster titled “Towards the increase of the thermostability of a psychrophilic enzyme”.

The oral and poster presentation awards were generously sponsored by the graduate research center of the University of South Wales, UK. Sam Nicholls, Danielle Avancini and Stefani Dritsa received F1000 awards for their presentations at the RSG UK symposium.

In addition to the aforementioned awards, HPC Wales chose Karen Sinu Ting’s work on “Concatabominations: identifying unstable taxa in morphological and phylogenomic supertrees using Safe Taxonomic Reduction” that enables Karen to get exclusive supercomputing access and support from the HPC Wales facility.

## Conclusion

The initiative of forming RSG UK and hosting the symposium was highly appreciated among peers, delegates and sponsors. The quality of student presentations and high profile keynotes were greatly admired. The participants agreed that the nature of the symposium and the scope of networking best suited their interest. Symposium organisers received positive feedback and comments from senior and junior researchers alike. Despite being organised by a newly formed group, with a very small initial membership, the attendance was excellent, which reflects that the news of the symposium was well disseminated. Overall, the symposium was a great success.

### Future editions

The successful completion and turn-around of the 1st symposium encouraged us to organise a yearly symposium. The RSG UK is pleased to announce that the 2nd student symposium will be organised for October 2015 and will take place at The Genome Analysis Centre (TGAC), Norwich. The aim is to arrange a symposium with a dedicated session for a bioinformatics workshop. Further details will be available on RSG UK website http://rsg-uk.iscbsc.org/.
